# The Efficacy of Isolated Bacteriophages from Pig Farms against ESBL/AmpC-Producing *Escherichia coli* from Pig and Turkey Farms

**DOI:** 10.3389/fmicb.2017.00530

**Published:** 2017-03-29

**Authors:** Aneta Skaradzińska, Paulina Śliwka, Marta Kuźmińska-Bajor, Grzegorz Skaradziński, Anna Rząsa, Anika Friese, Nicole Roschanski, Jayaseelan Murugaiyan, Uwe H. Roesler

**Affiliations:** ^1^Department of Biotechnology and Food Microbiology, Faculty of Food Science, Wrocław University of Environmental and Life SciencesWrocław, Poland; ^2^Department of Fermentation and Cereals Technology, Faculty of Food Science, Wrocław University of Environmental and Life SciencesWrocław, Poland; ^3^Department of Immunology, Pathophysiology and Veterinary Preventive Medicine, Faculty of Veterinary Medicine, Wroclaw University of Environmental and Life SciencesWrocław, Poland; ^4^Department of Veterinary Medicine, Institute for Animal Hygiene and Environmental Health, Freie Universitaet BerlinBerlin, Germany

**Keywords:** bacteriophages, antibiotic resistance, antibiotic resistance genes, ESBL/AmpC resistant *E. coli*, phage therapy

## Abstract

Extended-spectrum β-lactamases (ESBLs) and AmpC β-lactamases are plasmid (but also chromosomally) encoded enzymes found in Enterobacteriaceae, determining resistance to a variety of important antibiotics including penicillins, cephalosporins, and monobactams. In recent decades, the prevalence of ESBL/AmpC-producing bacteria has increased rapidly across the world. Here, we evaluate the potential use of bacteriophages in terms of a reduction of antibiotic-resistant bacteria in healthy animals. The aim of our studies was to isolate bacteriophages capable of destroying ESBL/AmpC-producing *Escherichia coli* isolated from livestock habitats. The efficacy of isolated phages against ESBL/AmpC *E. coli* strains varies, but creation of a phage cocktail with broad activity spectrum is possible. This may indicate that the role of phages may not be limited to phage therapy, but bacterial viruses may also be applied against spread of bacteria with antibiotic resistance genes in the environment. We also addressed the hypothesis, that phages, effective for therapeutic purposes may be isolated from distant places and even from different environments other than the actual location of the targeted bacteria. This may be beneficial for practical purposes, as the construction of effective phage preparations does not require access to disease outbreaks.

## Introduction

Extended-spectrum β-lactamases (ESBLs) and AmpC β-lactamases are plasmid and/or chromosomally encoded enzymes found in Enterobacteriaceae, conferring resistance to different β-lactam antibiotics, including penicillins, cephalosporins, and monobactams (Rawat and Nair, 2010). ESBL/AmpC-producing organisms are often co-, or multiresistant, exhibiting resistance to other anti-microbial classes including fluoroquinolones, aminoglycosides, and trimethoprim-sulfamethoxazole (EFSA Panel on Biological Hazards [BIOHAZ], 2011; Xu et al., 2015). To date the most common ESBL types are TEM, SHV, and CTX-M, which have been divided into five groups known as CTX-M-1, CTX-M-2, CTX-M-8, CTX-M-9, and CTX-M-25 (Bonnet, 2004). The most common plasmid-mediated AmpC type β-lactamase worldwide is CMY-2 (Jacoby, 2009). In veterinary medicine, ESBL-producing bacteria have been proved to cause mastitis in dairy cattle (Timofte et al., 2014), urinary tract infections (UTI) in companion animals (Keefe et al., 2010), and soft skin infections in companion animals and horses (Ewers et al., 2014). In humans, they may also cause UTI, pneumonia, or even sepsis (Peña et al., 2006; Falagas and Karageorgopoulos, 2009).

According to the European Food Safety Authority (EFSA), rapid emergence of resistance caused by ESBL in Enterobacteriaceae is a major public health concern in Europe (EFSA Panel on Biological Hazards [BIOHAZ], 2011). A report from the Infectious Diseases Society of America from 2006 listed ESBL-producing *Klebsiella* spp. and *Escherichia coli* among the six drug-resistant microbes for which new therapies are urgently needed (Pitout and Laupland, 2008).

In light of the growing threat associated with the spread of antibiotic resistance, the World Health Organization (WHO) and the European Commission (EC) have recognized the importance of studying anti-microbial resistance and highlighted the need to devise appropriate strategies for its control (Picozzi et al., 2014). Also the World Medical Association (WMA) appealed for increased research funding to develop new anti-microbial agents (Kelly et al., 2011).

Application of bacteriophages to combat bacterial diseases (phage therapy), despite its initial fast development, was significantly marginalized after the discovery of penicillin and other antibiotics (Sulakvelidze et al., 2001; Deresinski, 2009). Nowadays, a rapidly growing number of drug-resistant bacterial strains have already evolved as the most serious threats in public health. Therefore, phage therapy is recognized as one of the most attractive alternatives to classical antibiotic-based treatment and is becoming an important research objective.

Although phage therapy for the treatment of infections caused by antibiotic-resistant bacterial strains has been studied extensively (Biswas et al., 2002; Jensen et al., 2015), until now only a few studies have described the use of phages in terms of a reduction of antibiotic-resistant bacteria in healthy animals (Teng-Hern et al., 2014). In this study it was demonstrated that phages may be potentially applied to reduce the prevalence of ESBL/AmpC *E. coli* in healthy food-producing animals and thus may play an important role in the ecology of resistance. We also addressed the hypothesis that phages for this purpose can easily be isolated from distant places and even different environments than the targeted bacteria.

## Materials and Methods

### Environmental Samples

A total of nineteen samples were collected from six pig farms in Poland. Depending on the particular maintenance system (litter, deep litter, slatted floor), the samples were collected from bedding material (litter system) (*n* = 7), feces (slatted floor) – pooled or from individuals (*n* = 5), and feed taken from the feeders (*n* = 5). Additionally, two manure samples were collected from a manure lagoon.

### Isolation of Bacteria from Environmental Samples

From all collected environmental samples, bacteria were isolated and subsequently used for phage isolation and host range testing. Of each environmental sample (feces/litter/feed), 5 g were introduced into 30 ml of LB broth (Sigma–Aldrich, Germany) and incubated at 37°C for 20 h. On the following day, the overnight cultures were spread on Columbia Agar with sheep blood (BioMerieux, France) to obtain single colonies. All bacteria of different phenotypes were identified using the BBL Crystal Autoreader System (BD Biosciences, USA). In addition, the generic affiliation of bacteria to which specific phages were isolated was confirmed with matrix-assisted laser desorption/ionization time-of-flight mass spectrometry analysis (MALDI Microflex LT and Biotyper database, Bruker Daltonics, Bremen, Germany). Subsequently, bacteriophages were isolated from the same overnight cultures by filtration through 0.22 μm syringe filters (Merck Millipore, Germany).

### Isolation of Bacteriophages

The phage isolation was carried out using the modified method of Slopek et al. (1972). Each of the isolated bacterial strains was used to test for bacteriophage in each filtrate. Briefly, 5 ml of a 3-h incubated bacterial culture (LB broth) was poured out on a LB agar plate (excess culture was removed and the plate was left to dry) and 50 μl of each filtrate was introduced into each of eight sectors previously marked on the bottom of the plate. After drying of the drop, the plates were incubated at 37°C and plaque formation was monitored after 3, 4, 5, and 24 h.

### Amplification of Bacteriophages

Isolated bacteriophages were amplified in the respective host bacteria. Therefore, 20 μl of phage filtrate and 20 μl of the bacterial overnight culture were added to 10 ml of LB broth and incubated at 37°C. The optical density (OD_600_) of the culture was measured hourly. In case of an OD fall (phages eliminated most of the bacteria), additional host bacteria were added. If the OD increased (there were more bacteria in the culture than phages), additional phage filtrate was added. After a total incubation time of 6 h, all remaining bacteria were removed by filtration through a 0.22 μm syringe filter and the phage titer was determined using the double agar method (Adams, 1959). The experiments were performed in triplicate in order to eliminate bactericidal activity of some chemical compounds which might potentially be present in the sample.

### ESBL/AmpC-Producing *E. coli*

Extended-spectrum β-lactamases/AmpC-producing *E. coli* strains derived from the strain collection of the Institute for Animal Hygiene and Environmental Health, Freie Universitaet Berlin. The isolates were obtained from 7 different pig farms (*n* = 104) and 22 turkey farms (*n* = 51) in Germany. Each isolate originated from an individual sample including feces, boot swabs, manure, air or dust. ESBL/AmpC *E. coli* were selected using MacConkey agar (Oxoid, CM 0115, Wesel, Germany) supplemented with 1 mg/L cefotaxime, followed by species confirmation using MALDI-TOF identification (MALDI Microflex LT and Biotyper database, Bruker Daltonics, Bremen, Germany). The presence of the β-lactamase genes *bla*_CTX_, *bla*_TEM_, *bla*_SHV_ and the CIT-type AmpCs (e.g., CMY-2) was confirmed by real-time PCR as described by Roschanski et al. (2014).

### Bacteriolytic Activity of Isolated Bacteriophages against ESBL/AmpC *E. coli* Isolated from Selective Pig and Turkey Farms from Germany

One hundred four ESBL/AmpC *E. coli* strains originally isolated from pig farms from Germany and stored at the culture collections of the Institute for Animal Hygiene and Environmental Health, Freie Universitaet Berlin (Berlin, Germany) were chosen and tested for sensitivity to the 17 previously isolated bacteriophages. Additionally, 51 ESBL/AmpC *E. coli* isolated from turkey farms (from the same collection) were typed with the same phage preparations. The procedure was performed as described in the section Isolation of bacteriophages.

### Activity of Phage Cocktail against ESBL/AmpC *E. coli* Isolated from Pig Farms

Bacteriophages selected for the cocktail were mixed in the ratio 1:1:1 (V/V/V). The final titer of each phage was 1 × 10^9^ pfu/ml. The activity tests of the cocktail were performed as described for the tests of the monophage preparations.

## Results

### Occurrence of *E. coli* and *E. coli*-Specific Bacteriophages in Pig Environment Sampled in Polish Pig Farms

A total of 75 bacterial strains were isolated from the 19 collected samples including: *Proteus* spp. (22), *Escherichia* ssp. (out of 23 strains 20 were *E. coli*), *Aeromonas* ssp. (5), *Staphylococcus* ssp. (4), *Providencia* ssp. (3), *Klebsiella* ssp. (3), *Morganella* ssp. (2), *Citrobacter* ssp. (2), *Vibrio* ssp. (2), *Shigella* ssp. (2), *Serratia* ssp. (2), *Kytococcus* ssp. (1), *Corynebacterium* ssp. (1), *Acinetobacter* ssp. (1), *Aerococcus* ssp. (1), *Bacillus* ssp. (1). A total of 19 filtrates, one per sample, with potential bacteriophages were prepared and the sensitivity of all isolated bacterial strains tested against each bacteriophage. Twenty-two different bacteriophages, all specific to *E. coli*, were isolated. Three rounds of successive plaque purifications were performed to isolate each phage. Seventeen phages were successfully amplified for further studies.

### Activity of Isolated Bacteriophages against ESBL/AmpC *E. coli* Isolated from Pig Farms

**FIGURE 1:**
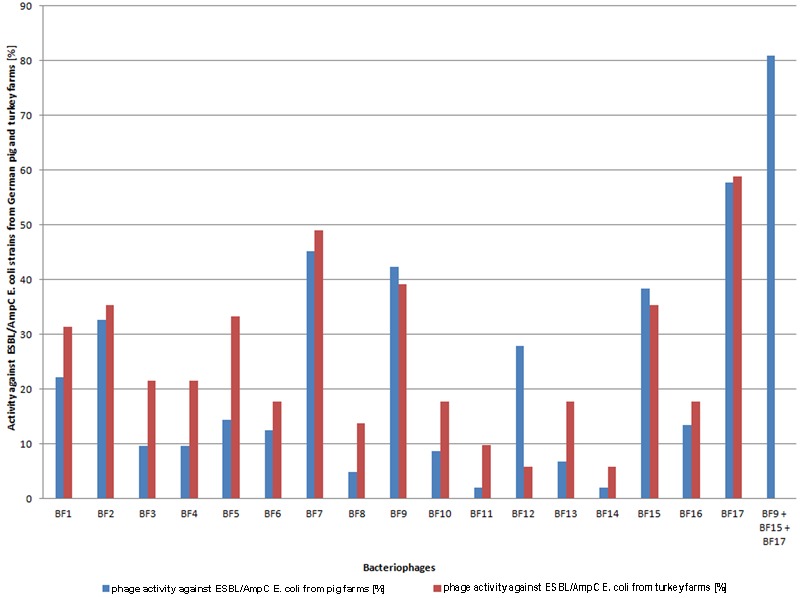
**Activity of bacteriophages isolated from Polish pig farms against ESBL/AmpC *Escherichia coli* isolated from German pig and turkey farms.** Blue and red columns mean activity (%) of bacteriophages isolated from Polish pig farms against ESBL/AmpC *E. coli* isolated from German pig and turkey farms, respectively, and the last column means activity (%) of a cocktail (BF9 + BF15 + BF17) against ESBL/AmpC *E. coli* isolated from German pig farms.

The activity of 17 bacteriophages isolated from Polish pig farms was tested against 104 ESBL/AmpC *E. coli* strains isolated from German pig farms. The results showed that all phages were active against the tested *E. coli* strains and they lysed from 1.9% up to 57.7% of all strains (**Figure 1**). 82.7% of all bacterial isolates were sensitive to at least one of the phages, which means that 17.3% were not sensitive to any of the phage preparations.

### Activity of Phage Cocktail against ESBL *E. coli* Isolated from Pig Farms

Based on the above results, a cocktail of three phages was prepared and tested against ESBL *E. coli*. The cocktail included phages BF17 and BF15, which showed the highest bactericidal activity. Phages BF7 and BF9 showed a similar activity against the tested *E. coli* strains (45.2 and 42.3%, respectively); however, to cover a broader spectrum of bacteria, for the cocktail we chose phage BF9 even though it showed slightly lower activity than phage BF7. The phages from hypothetical cocktail were characterized in terms of their morphology, genetics and phage-bacteria kinetics (data not shown). The selected cocktail lysed 80.8% of all tested strains.

To confirm the activity of the phage cocktail, nine ESBL *E. coli* strains with different sensitivity to the individual phages were chosen. The obtained results (**Table 1**) confirmed that the tested phage cocktail might potentially be useful against ESBL *E. coli*.

**Table 1:** Activity of bacteriophage cocktail (BF9 + BF15 + BF17) against selected Extended-spectrum β-lactamase (ESBL) *Escherichia coli* strains.

Bacterial strain	Origin	β-Lactamases	Previous observations	Obtained result (phage cocktail)
23	Single animal feces	CTX, TEM-1	No lysis caused by any of the phages	No lysis
35	Single animal feces	CTX	No lysis caused by phages from cocktail, but lysis caused by other phages	No lysis
36	Single animal feces	CTX	Active phages: BF15 + BF17	Lysis
80	Air sample (impingement)	CTX	Active phages: BF9 + BF15 + BF17	Lysis
86	Manure lagoon	CTX, TEM-1	Active phages: BF9 + BF17	Lysis
88	Pooled feces taken in the rearing quarter	CTX	Active phage BF15	Lysis
89	Boot swab inside the stable	CTX, TEM-1	Active phage BF17	Lysis
97	Single animal feces	CTX	Active phages BF9 + BF15	Lysis
103	Fly	CTX	Active phage BF9	Lysis

### Activity of Isolated Bacteriophages against ESBL/AmpC *E. coli* Isolated from Turkey Farms

To investigate whether the 17 phages may also be active against bacterial strains isolated from different animal farm environments, additional host range determination using 51 randomly selected ESBL/AmpC *E. coli* isolated from German turkey farms was performed. Interestingly, almost all phages (14/17) were more active against ESBL/AmpC *E. coli* isolated from turkey farms than ESBL/AmpC *E. coli* isolated from pig farms. Eleven out of the 51 strains were not sensitive to any of the phages, which means that the complete pool of phages lysed 78.4% of all tested strains (**Figure 1**).

## Discussion

The potential therapeutic applications of phages as one of the main alternatives to classical treatment have been widely explored. Bacteriophages have already been successfully applied in treatment of antibiotic-resistant bacterial strains, including methicillin-resistant *Staphylococcus aureus* (Jensen et al., 2015) and vancomycin-resistant *Enterococcus* sp. (Biswas et al., 2002). In addition, the effectiveness of phage ø9882, isolated from hospital sewage, against a broad range of ESBL-producing *E. coli* clinical isolates has been shown in mice. It is worth emphasizing that the studies of Wang et al. (2006) provided a framework to evaluate the therapeutic efficacy of phages against fatal ESBL *E. coli* infections in humans (Wang et al., 2006). However, our research shows that the role of phages may not be limited to phage therapy, but bacterial viruses may also be applied against spread of bacteria with antibiotic resistance genes in the environment.

In the first part of the present study, a total of 17 bacteriophages specific to *E. coli* were isolated from Polish pig farms. Phages were isolated only with bacteria simultaneously isolated from the same environment. In the second stage of the study, the bactericidal activity of phages isolated from Polish farms was evaluated against unrelated bacterial isolates (ESBL/AmpC *E. coli* isolated from German pig farms). All phages were found to be active against some of the tested strains, but they lysed these strains with different efficacy.

Possible activity of the isolated phages against ESBL/AmpC *E. coli* from animal species other than pigs was also investigated. However, it is worth noting that the tested strains isolated from pig and turkey farms were not compared genetically, and thus it is theoretically possible that the same bacteria were isolated from both environments. Interestingly, almost all phages showed higher or similar activity against the strains originating from turkey compared to the pig isolates.

In the past, the focus of most phage therapy studies lay in finding phages against specific pathogens. This was performed by on-demand isolation methods in which targeted bacterial strains were added to the culture to enrich potential bacteriophages. This seems to be highly effective, since it was successfully done with phages against *Pseudomonas aeruginosa* and *Salmonella* but also against ESBL *E. coli* and *Klebsiella* (Mattila et al., 2015). In this study, we addressed the hypothesis that phages, isolated from distant places and even deriving from different farm environments than the targeted bacteria, may also be suitable for an efficient polyphage therapy. This is of great importance, as it proves that the construction of effective phage preparations does not require access to disease outbreaks.

Interestingly, the relationship between the presence of phages and sensitive bacteria has already been observed at the stage of phage isolation. In an initial spot tests we selected 22 potential bacteriophages for purification (17 phages were successfully amplified for further studies). As we analyzed the origin of these 22 phages and their hosts, 18 phages were active against bacteria from different farms. Furthermore, to compare the effectiveness of the on-demand method with isolation of phages from distant locations and environments, we additionally prepared phage filtrate obtained using this technique (Supplementary Figure 1). To create this preparation, we used the same 51 *E. coli* strains from turkey farms, which were used for activity tests of phages from pig farms. Phage filtrate obtained using the on-demand method lysed 80.4% of all tested strains. To compare, three of 17 phages from Polish pig farms (BF5 + BF15 + BF17) lysed this pool of bacteria with only slightly lower efficacy of 78%. It is noteworthy that we did not separate individual phages from the preparation obtained using the on-demand method, but while testing the activity of phages against bacteria we used the whole filtrate, which may hypothetically contain several different phages. We may assume that after isolation of individual phages it would not be possible to create a 3-phage cocktail with similar efficiency as the one from pig farms.

The results of this study confirmed previous observations of Jensen et al. (2015), who demonstrated that phages active against human methicillin-resistant and methicillin-susceptible *S. aureus* may be easily isolated from chicken sources, which are not necessarily predicted to be a good source of phages against these bacteria. Notably, in contrast to our studies, these phages were isolated using the on-demand method. Gutiérrez et al. (2016) found no clear association of infectivity of phages against *S. aureus* strains based on the geographic region of host or phage isolation. However, the authors postulated a possible association between phage infectivity and the environment. This was also mentioned by Vos et al. (2009), who suggested that phages may adapt to become more effective against bacteria living closely.

Although person-to-person spread is recognized as the main method of dissemination of ESBL/AmpC bacteria in the community, the prevalence of ESBLs nowadays has increased significantly due to *E. coli* isolates from food-producing animals (Carattoli, 2008; Geser et al., 2012; Samanta et al., 2015). Furthermore, the ESBL/AmpC-producing *E. coli* in animal farms could influence public health through environmental pollution (Li et al., 2015). Fecal emission and the airborne route might be possible ways of ESBL/AmpC emission into the environment (Yuan et al., 2010; Friese et al., 2013; Laube et al., 2014). Moreover, vectors such as flies (Usui et al., 2013) or small rodents (Kozak et al., 2009) may also spread anti-microbial resistance. The landform may also boost the dissemination of bacteria, as they may be spread with the rainfall from farms situated in the hills. In addition, if antibiotics are used in the farms, residues can enter the waterways and be transferred to distant locations, creating an antibiotic resistance problem in new locations. As bacteriophages may be transmitted in the environment via similar pathways, the spread of phages and host bacteria may have a wide range. This may be one explanation why active phages may be isolated from distant locations and different environments than targeted bacteria.

Another possibility is that phages become lysogenic during transmission in the environment and they revert back to lytic ones at distant places. They may originate from farms other than the one from which they were isolated.

Higher activity of phages from distant farms and environments than in the case of co-isolates may be due to the occurrence in different environments and the lack of direct contact that would result in the development of resistance to phages, as observed in phage-treated campylobacteriosis (Loc Carrillo et al., 2005). The phenomenon of bacterial phage resistance is, however, complex and probably depends on the phage-bacteria configuration and time points of interactions occurring between bacterial viruses and their hosts (Fischer et al., 2013). The data regarding phage resistance are scarce and contradictory; thus detailed studies to explain this phenomenon need to be conducted.

Although the results of this study indicate the relation between the geographic distance and type of environment from which active phages are isolated, they are not sufficient for the conclusion that a particular source is better for the isolation of effective phages. Studies including the use of samples from several different environments and locations would be more reliable, especially considering the contradictory data in the literature.

The present study indicated that bacterial viruses have a potential for prevention or treatment of *E. coli* with ESBL genes. Moreover, they may be good candidates in control strategies against the widespread presence of ESBL/AmpC *E. coli* in healthy food-producing animals and hence the reduction of these bacteria in the entire food chain. Phages for this purpose may be easily isolated from the environment, but the question whether distant locations and different environments are better sources of active phages remains open. Although phages are considered one of the least known entities on our planet (Grose et al., 2014) and further studies regarding their biology need to be conducted, they undoubtedly could provide a novel approach to the prevention and treatment of infections caused by drug-resistant bacteria.

## Author Contributions

AS devised the experiments, acquired and processed laboratory data and wrote the manuscript, PS, MK-B, and GS performed the experiments related to isolation of bacteriophages, AR collected the environmental samples for isolation of bacteriophages, AF and NR designed the studies related to phage activity against ESBL/AmpC *E. coli*, JM confirmed the generic affiliation of bacteria with MALDI TOF mass spectrometry analysis, UR designed the studies and examined the manuscript. All authors participated in writing the manuscript and approved its final version.

## Conflict of Interest Statement

The authors declare that the research was conducted in the absence of any commercial or financial relationships that could be construed as a potential conflict of interest.
