# Supplementary figures and images for: The Efficacy of Isolated Bacteriophages from Pig Farms against ESBL/AmpC-Producing Escherichia coli from Pig and Turkey Farms
Source: Front Microbiol. 2017 Mar 29;8:530. doi: 10.3389/fmicb.2017.00530 (PMC5370273; doi:10.3389/fmicb.2017.00530)

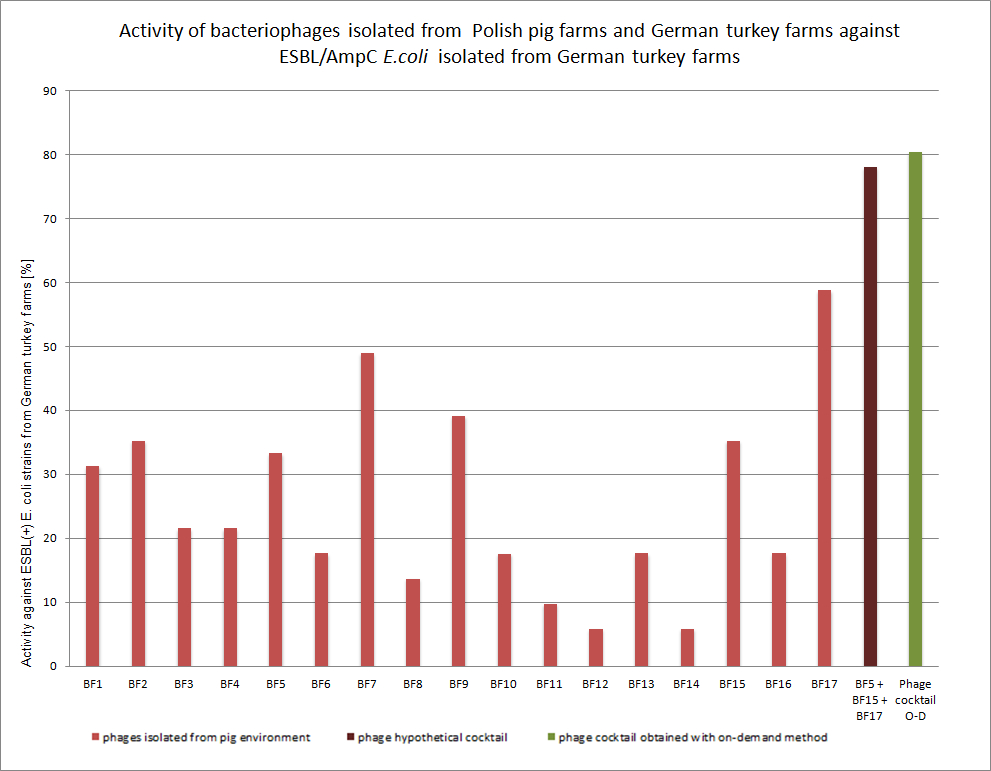

Supplement: Supplementary file 1 [file Image_1.JPEG]
